# Urban Transformations to Keep All the Same: The Power of Ivy Discourses

**DOI:** 10.1111/anti.12820

**Published:** 2022-03-08

**Authors:** Linda Westman, Vanesa Castán Broto

**Affiliations:** ^1^ Urban Institute University of Sheffield Sheffield UK

**Keywords:** urban transformations, urban sustainability, discourse, radical theory, cities

## Abstract

The concept of urban transformations has gathered interest among scholars and policymakers calling for radical change towards sustainability. The discourse represents an entry point to address systemic causes of ecological degradation and social injustice, thereby providing solutions to intractable global challenges. Yet, so far, urban transformations projects have fallen short of delivering significant action in cities. The limited ability of this discourse to enable change is, in our view, linked with a broader dynamic that threatens progressive commitments to knowledge pluralism. There are discourses that, cloaked in emancipatory terminology, prevent the flourishing of radical ideas. The ivy is a metaphor to understand how such discourses operate. Ivy discourses grow from a radical foundation, but they do so while reproducing assumptions and values of mainstream discourses. We are concerned that urban transformations functions as an ivy discourse, which reproduces rather than challenges knowledge systems and relations that sustain hegemony.

## Resumen

El concepto de transformaciones urbanas ha ganado interés entre académicos y profesionales que piden un cambio radical hacia la sostenibilidad. Este discurso representa un punto de entrada para abordar las causas sistémicas de la degradación ecológica y la injusticia social, proporcionando soluciones para los desafíos globales que afrontan las ciudades. El entusiasmo teórico por proyectos de transformación urbana, sin embargo, no se corresponde con sus impactos prácticos que, de momento, han sido limitados. Las limitaciones del discurso transformativo son, en nuestra opinión, síntomas de un problema más amplio relacionado con la dificultad para integrar una visión pluralista del conocimiento en la política urbana. Hay discursos que, disfrazados de terminologías de emancipación, impiden el florecimiento de ideas radicales. Llamamos a estos discursos discursos‐hiedra, donde la hiedra es una metáfora que explica cómo funcionan. Los discursos‐hiedra crecen a partir de una base radical, pero lo hacen mientras reproducen las premisas y valores de discursos dominantes. Cuando el discurso de transformación urbana funciona como discurso‐hiedra, sirve para reproducir las estructuras de poder hegemónico, en lugar de transgredirlas.

## Introduction

Boaventura de Sousa Santos (2015) has argued that academic‐activists need *untraining* in forms of thinking and theorising that he calls vanguard theory. This is a form of thinking that explains everything in advance, thus excluding any form of knowledge or experience that does not fit its prescriptions. As an alternative, Santos advocates for rearguard theory, which actively engages with the affective, situated experiences that shape knowledge. An essential task for academic‐activists is, thus, the creation of workable discourses that recognise the multiplicity of dislocated experience in a world shaped by rampant inequality and a global environmental crisis.

New concepts and ideas can generate momentum, opening such spaces of plural engagement and supporting moves toward more hopeful futures where difference is recognised. Brown (2016), for example, followed Laclau (1990, 2005) in describing sustainability as an empty signifier whose potential resides in its multiple interpretations and contingency of meaning. This analysis portrays sustainability as a discourse that offers radical potential because it enables ever‐more diverse critiques of the economic system and brings sustainability debates into the realm of politics. Engagement with sustainability discourses, however, has declined in recent years.

The discourse of transformations appears to have displaced sustainability (Blythe et al. 2018). Transformations‐oriented policy is seen as bridging the chasm between the severity of environmental problems and the inherent incrementalism of existing environmental policy (Abson et al. 2017). Transformation processes have become the raison d’être in the growing literature on sustainability and development (Scoones 2016). Beyond the delivery of deep change to protect ecosystems, transformations represent a new normative agenda for social justice (Patterson et al. 2018). The transformations vocabulary has undergone a rapid translation from scholarly debates into multiple policy settings. For example, the United Nations Agenda 2030 for Sustainable Development is promoted under the banner of “Transforming Our World”, guided by “a supremely ambitious and transformational vision” (United Nations 2015). Despite the generalised enthusiasm, the concept of transformation also appears to be linked with risks, especially the further depoliticisation of environmental discourses (Blythe et al. 2018). Is transformation a new “empty signifier”, in the sense articulated by Brown (2016)? Does it lead to obliteration of difference (Blythe et al. 2018), or does it open spaces for Santos’ (2015) rearguard theory?

This paper examines how discourses of transformation influence academic‐activists’ role in climate policy. The analysis focuses on urban transformations, a domain particularly visible in climate change and transformation agendas. Urban transformations have become the foremost entry point to address global environmental challenges and maintain hope. The New Urban Agenda, adopted in Quito in 2016, framed urbanisation as an opportunity to achieve “transformative and sustainable development” (United Nations 2016). The IPCC Special Report on Climate Change of 1.5 degrees included a section dedicated to urban transformations, noting that “[l]imiting warming to 1.5° C above pre‐industrial levels would require transformative systemic change” (de Coninck et al. 2018:315). Among the many high‐profile reports launched at COP26, the Coalition for Urban Transitions published a report on “Seizing the Urban Opportunity”. The explicit purpose of this document was to demonstrate “the power” of urban transformations to catalyse national‐level recovery from the COVID‐19 pandemic, while also tackling the global environmental crisis (Coalition for Urban Transitions 2021).

In this paper, we examine how urban transformations discourse has gained credibility and, in doing so, may reproduce dominant research and policy agendas and consolidate epistemic centres of authority. We argue that there is a specific mechanism at play behind this success story, which we characterise through the idea of *ivy discourses*. Ivy discourses operate much like the plant ivy (*Hedera helix*): the ivy grows around a solid foundation with intense vigour, developing shining leaves that soon cover the supporting plant. The ivy can conceal the structure that sustains its existence and growth, on occasions consuming the supporting structure—the conceptual apparatus that eventually crumbles, dies, and is forgotten under its crushing weight. We ground the concept of ivy discourse in the discussion of empty signifiers, following Laclau (2005) and Brown (2016). We hypothesise that urban transformations theory is an exemplar of an ivy discourse. The attraction of transformations lies precisely in their promise to deliver radical change. Yet, there are risks inherent in mobilising putatively radical concepts with limited ability to support emancipatory thought and action.

Our analysis opens questions about the desirability of transformation as a radical concept, particularly in comparison with its predecessor, sustainability. First, we explain why we focus on discursive elements of urban transformations and how to interpret the concept in dialogue with the notion of the empty signifier. Second, we outline the conceptual foundations of transformations theory. Next, we discuss the articulation of urban transformations theories as an ivy discourse. The analysis identifies the mechanisms that have enabled the consolidation of the theory in research and practice. To illustrate the tendency of “concealment”, we compare trends of thought in urban transformations debates with principles for social change derived from decolonial and feminist thought. In conclusion, we suggest that the search in academia for compelling and radical answers may lead to the reproduction of hegemonic ideas and interrogate whether rearguard alternatives are available.

## A Discursive Approach to Transformations

Bluwstein (2021) recently proclaimed that transformations is not a metaphor. Scholars of transformations, he argues, spend too much time examining epistemologies, ideologies, and other abstract dimensions. What is needed instead is engagement with practical aspects of change: strategy, organisation, and tactics to dismantle political economic structures. This argument is refreshing and critically needed. However, the impact of discourses cannot be discounted. Discourses structure repertoires of action and charter directions of change. They are inseparable from political interventions.

Discourses represent systems of meaning ascribed to objects and events. While meaning can be communicated through language, discourses also condition interpretations of other forms of actions and interactions, such as sense‐making in relation to material artefacts (Wetherell et al. 2001). By organising social relations, practices, and relationships with technology, discourses dictate conduct (and our interpretations of that conduct) in everyday life. In political analyses, discourses also represent power relations and systems of rule that are historically constituted (Howarth et al. 2000). If the social is a discursive space, hegemonic formations are constituted through contingent conditions, themselves dependent on existing discourses (Laclau and Mouffe 2001). In the context of environmental transformations, discourses reinforce forms of symbolic violence that justify environmental destruction in the face of manifest injustices towards people and nature (Castán Broto 2013). At the same time, the very notions of cultural hegemony, symbolic violence, and contingent strategy are part of a Eurocentric tradition of thought suspicious in decolonial scholarship (Grosfoguel 2011). By examining discourses, we seek to engage with an active critique of transformations not as detached critical observers, but as knowledge producers who have actively contributed to that scholarship.

Hence, this paper focuses on the relationship between hegemonic discourse and concepts that (seem to) represent radical alternatives. Different theories that explain this connection have inspired our conceptualisation of ivy discourses. For example, scholars of sociology of science (Davis 1986) identify features of academic concepts that explain their ability to gain social influence. There is a category of fuzzy social science concepts that gain popularity precisely by virtue of being vague, which means that they are open to contestation (Cornwall 2007). Davis (2008), for instance, argues that the concept of intersectionality gained traction far beyond feminist theory by being open‐ended and ambiguous, but also by addressing a pervasive and serious concern, providing novelty, and appealing to generalists. Transformations theory fits this description. It is unceasingly open‐ended (allowing for appropriation), addresses a serious concern (threats to the survival of humanity), provides novelty (through the emphasis on system‐wide change), and appeals to generalists (striking an elegant balance between sophisticated diagnostics and easily derived principles). This sheds light on the success of the transformations theory, but leaves us with questions: what normative implications do buzzwords raise (Durose et al. 2022) and what is their relation to hegemonic discourses?

To gain clarity on mechanisms of discursive hegemony we follow Laclau (2005:68), who defines discourse as “any complex of elements in which *relations* play the constitutive role”. Laclau argues that the differentiated demands of social groups somehow must be unified to act as a political whole. Such unification is realised by reducing a plurality of demands to a chain of “equivalents” (demands that are equal in relation to the totality) and allowing these demands to be represented by empty signifiers (totalities that reflect an abstract ideal of unachievable perfection). Hegemony operates through empty signifiers by enabling a particularity to achieve “incommensurable universal signification” (Laclau 2005:70).

Environmental discourses that represent progressive solutions are routinely appropriated, which enables the reproduction of dominant paradigms (Castán Broto and Westman 2019). This openness invites analysis along Laclau’s notion of empty signifiers. For example, Gunder (2006:214) argues that sustainability:

acts as an empty name or label of an ideal that many can believe and identify with. Yet, in doing so, sustainability accommodates a wide range of contestable discourses, each vying to articulate its definitive meaning.

This critique aligns with a consolidated scholarship on sustainability, which points to ambiguity and open‐endedness as features that enable the advance of dominant political‐economic interests (De Lara 2018; Lélé 1991; Manderscheid 2012). At the same time, the capacity of empty signifiers to resist the attribution of unitary meaning make them useful to social groups. Brown (2016:129) posits that sustainability behaves as an empty signifier, not only in the sense of being empty of environmental content, but also because it “represents the imagined fullness of society that is (presently) absent”. Brown (2016) argued that sustainability, as an empty signifier, created radical political momentum by foregrounding a collective failure to think about the future. By representing a response to diverse apocalyptic imaginaries, sustainability succeeded in building political unity, as well as in shifting debates from an interest in piecemeal intervention towards notions of systemic change. In operating as an empty signifier, sustainability thereby enabled radical action, by accommodating an increasingly diverse range of critiques and re‐politicising sustainability debates (Brown 2016).

Laclau (2005) explains that oppressive regimes build hegemonies by claiming empty signifiers. This is achieved by articulating new links around popular demands, with the result that the “*same* democratic demands receive the structural pressure of *rival* hegemonic projects” (Laclau 2005:131). As the meaning of a signifier becomes “indeterminate between alternative equivalential frontiers”, empty signifiers transform into floating signifiers, the meaning of which can only be determined by (hegemonic) struggle (ibid.). Political resistance always involves heterogeneity, meaning that there are no immobile frontiers along which empty signifiers can be clearly defined. As heterogeneous communities lay claim on a concept definition, they progressively multiply frontiers of struggle. It is in this moment that the potential for both radical and hegemonic appropriation emerges.

Our argument is that ivy discourses appropriate signifiers and help control their radical potentialities. What Laclau (2005) describes as the articulation of new links around demands manifests through the coupling between radical visions and established patterns of thought. Specifically, concepts become decentred from emancipatory aims and struggles by becoming attached to pre‐existing disciplinary knowledge, normative ideals of societal improvement and progress, and theoretical apparatuses. This crowds out the discursive space and places original demands in a minority position that eventually becomes invisible.

## The Foundations of Transformations Theory

Transformations theory is a solutionist approach that proclaims to tackle environmental degradation and deliver social justice. In practice, two literature bodies have dominated transformations research: socio‐ecological systems and socio‐technical transitions studies (Table 1). Both literatures address systems reconfigurations, but the former focuses on how social‐ecological systems cope with disruptive change and the latter on nonlinear changes in socio‐technological systems (Olsson et al. 2014:1).

**Table 1:** The theoretical foundations of transformations studies

Theoretical foundation	Key concepts	Process	Outcomes
Socio‐ecological systems (SES)	Resilience, adaptive capacity, adaptive management, institutions	As conditions become untenable, a socio‐ecological system undergoes fundamental reconfiguration through establishment of new human–nature interactions	New relationships between species populations and system variables, new adaptive cycles, new feedback mechanisms and new institutional arrangements
Socio‐technical systems	Socio‐technical regimes, co‐evolution, niches, landscapes	Interaction between niche innovation and landscape pressures, co‐evolution between multiple elements of technology and society	New rule sets and new social‐technical alignments (shifts in culture, markets, policy, industry, and science)

Social‐ecological systems (SES) studies have a strong basis in ecology and systems theory and use resilience and adaptation as their fundamental building blocks. Holling (1973:14) introduced resilience as a concept that measures the ability of a system to remain in the same state, that is, its ability to preserve relationships between species populations and system variables. This concept challenged the notion of stable equilibria and inspired engagement with unpredictability and random events. Adaptive capacity describes the “change in stability landscapes” that occur in an ecosystem in response to ecosystem pressures (Gunderson 2000:428). Adaptive capacity is shaped by external stresses that determine exposure and sensitivity and the interplay of social, economic, and political forces within a given system (Smit and Wandel 2006).

Building on this thinking, Walker et al. (2004:3) defined a transformation as the “capacity to create a fundamentally new system when ecological, economic, or social (including political) conditions make the existing system untenable”. A transformation occurs when new human–nature interactions are established, alongside new adaptive cycles, feedback mechanisms, and governance arrangements (Olsson et al. 2014; Walker et al. 2004). “Transformability” of a social‐ecological system depends, in turn, on various properties of a system, such as human resources, institutions, and capacity for cross‐scale interactions (Walker et al. 2004).

Social‐ecological systems perspectives take ecological theory as their point of reference. However, they integrate insights from economics and institutional theory to understand “the source and role of change in systems—particularly the kinds of changes that are transforming” (Gunderson and Holling 2002:5). For example, adaptive management is an approach that “acknowledges that the natural resources being managed will always change, so humans must respond by adjusting and conforming as situations change” (Gunderson 2000:433). Adaptive forms of governance involve flexibility, collaboration and social learning, self‐organisation and polycentricity, trust and knowledge, bottom‐up participation, and deliberation, all unfolding across multiple scales of action (Lebel et al. 2006; Olsson et al. 2004). Transformative governance, similarly, involves experimentation and social learning (Turnheim et al. 2018).

Socio‐technical transitions research follows a tradition of evolutionary thinking in innovation studies and allied disciplines interested in interconnected processes of technological and societal change. Socio‐technical transitions theory draws on history and sociology of technology (Bijker et al. 1987) and innovation economics (Nelson and Winter 1977) to explain how technologies are embedded in multiple domains of society. The interconnected set of elements that enable the provision and use of a certain technology constitute socio‐technical “regimes”. For example, using a car requires a set of road infrastructures, maps, circulation rules, perceptions of car desirability, and consensus on the relations between cars and other vehicles, just to mention some components of that socio‐technical regime. The regime consists of semi‐coherent rules and institutions that provide a stable framework to organise relationships between markets and industries, socio‐cultural norms and practices, policy frameworks, and scientific paradigms (Geels 2004). The transitions literature explains that system change requires reconfiguration of these interconnected elements through the interaction between a diverse set of actors and co‐evolutionary development between technology and society (Elzen et al. 2004). Transitions research has examined in detail the governance arrangements that enable systems reconfigurations. For instance, transition management is a policy‐oriented field that engages with management strategies for complex systems dynamics. This branch of the literature has emphasised the capacity of policymakers to stimulate protected innovation environments, build long‐term visions, construct coalitions around transition agendas, adopt flexible goals, and adjust these regularly in line with societal learning (Kemp et al. 2007).

Cross‐fertilisation between the scholarships on transformations and transitions is common, and despite their different origins the concepts are today used in parallel (McPhearson et al. 2016) or even interchangeably (Wolfram et al. 2016). Deliberate attempts to combine the approaches, such as in social‐ecological‐technological systems approaches (SETS), have mapped the complexities involved in multiple, simultaneous transformations (Egerer et al. 2021). Both socio‐technological and socio‐ecological analyses pay attention to the multi‐layered structure of complex systems, propose action on multiple scales, emphasise nonlinear and unpredictable change, and interdependence of pathways. A systems focus is the fundamental ontological assumption that brings them together.

Radical elements have been part of the debate in both traditions of thought. On the one hand, there are efforts to progressively incorporate political theories and justice concerns into this research. Recent debates on transformative action to address climate change, for example, emphasise the need to protect vulnerable groups from the impacts of transformations and to enhance opportunities for inclusive decision‐making processes (Patterson et al. 2018). Transition studies originally displayed a relatively bounded interest in socio‐technical interactions, which attracted criticism regarding weak conceptualisations of politics and power (Shove and Walker 2007). As transitions are linked to visions of the future, their inherently normative natura has become apparent (Meadowcroft 2009; Smith and Stirling 2010). More recently, environmental justice insights have been explored in discussions of “just transitions” (Heffron and McCauley 2018). Just transitions perspectives involve examining ecological and social benefits and burdens, as well as aspects of inclusion, power, and recognition (Newell and Mulvaney 2013; Swilling et al. 2016).

Moreover, there are branches of the transformations literature that place radical ideas at their core. Within the literature on resilience and adaptation, transformations represent a force that can fundamentally restructure social institutions, political systems, and power structures. Pelling (2010:3) views transformative adaptation as “an opportunity for social reform, for the questioning of values that drive inequalities in development and our unsustainable relationship with the environment”. This presents transformations as processes that challenge the root causes of environmental destruction, such as “the macro‐economic growth paradigm of modernising development discourses” (Pelling et al. 2015:125). O’Brien (2018) similarly speaks of transformations as change that involves not only new technologies and behaviours, but also reorganisation of institutions and profound shifts in belief systems. Because global environmental change is embedded in deep structures of society, Nightingale et al. (2020) argue that transformations imply nothing less than the scrutiny of science itself and a wholesale reimagination of human–nature relations. To others, transformations present an opportunity to reform global economic systems and challenge the normative attachment to economic growth (Göpel 2016) or to “development” (Escobar 2015). In summary, the scholarship on transformations occupies a range of ideas, from proposals that locate change from within existing systems to approaches that challenge the fundamentals of society.

## The Operation of Urban Transformations Theory as an Ivy Discourse

If radical change is central to transformations, why is the discourse failing to advance radical ideas? Blythe et al. (2018) refer to a “dark side” of transformations to describe the risks involved in its establishment, especially perils involved in erasing resistance and conflict. They observe that:

[T]he dark side of transformation, by which we mean the risks associated with discourse and practice that constructs transformation as apolitical, inevitable, or universally beneficial, has the potential to produce significant material and discursive consequences. (Blythe et al. 2018:1218)

We agree with this critique, but we have also witnessed significant efforts to ameliorate these limitations. As described above, the scholarship on transformations has become more political and tuned towards just outcomes. Many perhaps experience a sense of déjà vu regarding critiques from decades of sustainability research and the by‐now well‐established responses: make processes more inclusive, collaborative, and reflexive. Ultimately, the suspicion remains that the discourse somehow fails to empower emancipatory thought.

The operation of ivy discourses help us understand why this is the case. Below, we elaborate on three mechanisms through which ivy discourses influence research and policy. First, ivy discourses reproduce dominant research agendas by linking core definitions and themes with pre‐existing programs of investigation. Second, ivy discourses align new fields of research with established notions of social improvement and progress, thereby cementing their underpinning values. Third, ivy discourses attach nascent ideas to pre‐existing heuristics, contributing to the consolidation of epistemic centres of authority.

For each mechanism, we illustrate our argument by outlining how urban transformations research fails to engage with structural drivers of ecological exploitation and inequality, as identified by feminist and decolonial scholarship. Rather than presupposing characteristics that make a theory radical, we rely on these literatures to demonstrate how ivy discourses routinely invisibilise “minor” theory. This represents a form of scholarship that aspires to develop theoretico‐practical connections (Katz 1996) and that resonates with what Santos (2015) calls rearguard theory—an alternative to any universalising dogma. In that context, the decolonial scholarship draws attention to the legacies of an imperial world order and the resulting systems of oppression (e.g. Grosfoguel 2011; Mignolo 2017), while the feminist literature challenges domination that results from patriarchy and other forms of othering (e.g. hooks 2000). There is a need for theoretical work that engages with the material aspects of environmental degradation and resource exploitation, proposing new discourses that enable local radical responses with global purchase (e.g. Obeng‐Odoom 2020). However, there is also a need for recognising a plurality of perspectives and values, such as we advocate here.

**Figure 1:**
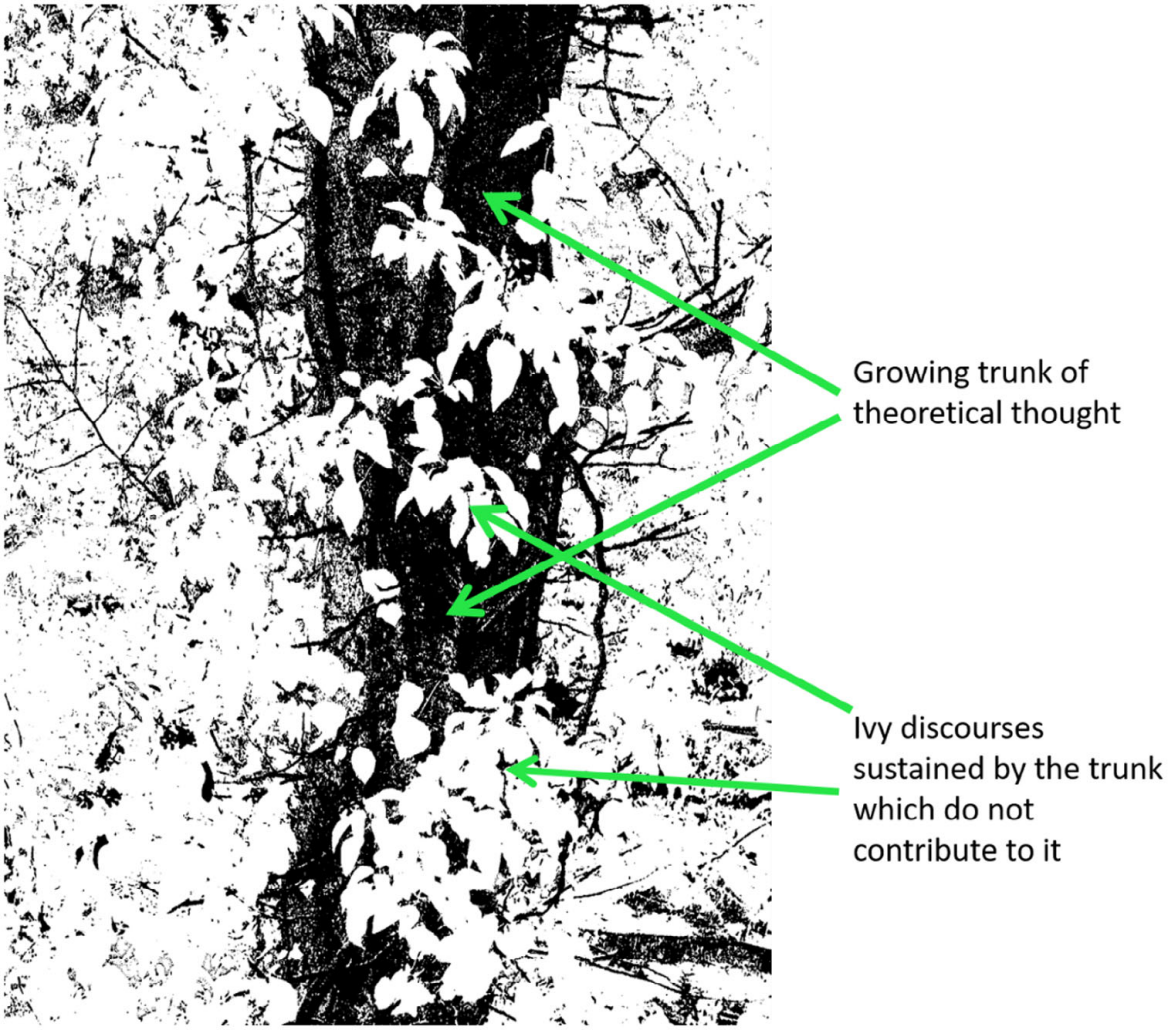
The operation of ivy discourses (source: authors) [Colour figure can be viewed at wileyonlinelibrary.com]

Table 2 and Figure 1 provide a summary of our critiques, expanded in the discussion below.

**Table 2:** The operation of urban sustainability transformations as an ivy discourse

Operation of ivy discourses	Feminist/postcolonial perspectives	Summary of critique
Reproduction of dominant research agendas through core definitions and themes	Challenging and deconstructing social categories that maintain inequality	Urban transformations research focuses on governance arrangements that enable resource efficiency and infrastructure optimisation. Yet, there is limited or no engagement with systems of discrimination, identity formation, and the othering of groups of people or nature based on social categories of difference
Alignment with social aims and notions of progress that cement underlying norms and values	Addressing the inequalities produced through the capitalist economic system	Urban transformations research displays concern with path dependencies and system lock‐ins, but rarely aims to challenge the structure of the world economy. There is little or no effort to challenge the sources of amassed wealth, growth‐oriented policy, or the organisation of global markets
Attachment to pre‐existing frameworks and consolidation of epistemic sources of authority	Tackling distortions in processes of knowledge production	Urban transformations research shows an interest in knowledge co‐production, but it is not sufficient to overcome the contradictions embedded in communicative rationalities. Eurocentric theoretical frameworks dominate the debate because academic communities are embedded in a hierarchical international system of knowledge production. There is a limited engagement with multiple epistemologies or situated action

### Reproduction of Dominant Research Agendas

Urban transformations theory acts as an ivy discourse by importing established problem frames and agendas from research on urban planning, sustainability science, environmental management, and similar fields, which causes perspectives within dominant disciplines (e.g. engineering, systems design, economics) to occupy the centre of debates. These disciplines readily propose solutions known to them and familiar to policymakers (e.g. resource management, efficiency upgrades, quantification of nature), which become presented as solutions to deep problems in society.

This tendency is visible in how transformations are defined in the context of urban sustainability. An urban transformation is, according to Elmqvist et al. (2019:269), a

systemic change of the urban system. It is a process of fundamental irreversible changes in infrastructures, ecosystems, agency configurations, lifestyles, systems of service provision, urban innovation, institutions and governance.

This definition prefigures a set of dimensions as central to transformations, including infrastructures, ecosystems, and lifestyles, which readily link to analyses in environmental planning, socio‐ecological systems, and economics. According to another study, key dimensions of sustainable urban transformations include governance systems, technology and innovation, lifestyles and consumption, infrastructure, and the built environment (McCormick et al. 2013). As above, this focus maps onto well‐established research in the policy and management literatures, innovation studies, and engineering. A third proposition is that urban transformations depend on ecological infrastructure, a mixture of land‐use types, reduced waste, integrated planning, and effective governance (Pickett et al. 2013). This, again, draws attention to recognised dimensions of environmental planning and resource management.

These concept definitions map onto research agendas fixed largely in studies on urban planning and design. The case studies that proliferate foreground issues such as the management and design of systems of energy (Olazabal and Pascual 2016), mobility (Hodson et al. 2017), water (Rijke et al. 2013), waste (Uyarra and Gee 2013), regeneration of districts (Block and Paredis 2013), spatial planning (Ernst et al. 2016), and interventions in the built environment (Vergragt and Brown 2010; Williams 2016). These case studies centre around themes that have dominated environmental studies for decades, such as how to design infrastructure systems and spatial plans to realise resource conservation. This focus also translates into a strong interest in resource efficiency (Kabisch and Kuhlicke 2014; Koch et al. 2016; Krellenberg et al. 2016), natural resource management (McCormick et al. 2013), and ecosystem services (Hansjürgens et al. 2018; Krellenberg et al. 2016; Li et al. 2018).

Comparing this focus with proposals for change towards social justice and environmental integrity in decolonial and feminist scholarship reveals aspects that are absent. An overarching concern in these literatures is to challenge and deconstruct social categories that maintain relations of domination. Santos (2015) describes the world order in relation to a global abyssal divide. Those who are excluded suffer dispossession and violence; those who are included are often numb to this reality. Colonisation established many of the racialised categories that normalise this divide (Grosfoguel 2002; Quijano 2007; Said 1978). Racism serves to de‐humanise some groups of people and reproduces conditions that leave them without rights, material resources, or social recognition (Grosfoguel 2016). Structural racism permeates public institutions, legal systems, and many expressions of state violence (Jee‐Lyn García and Sharif 2015; Saito 2009). Gendered and sexual inequalities also perpetuate the abyssal divide in explicit acts of violence and exploitation, as well as in subtle systems of repression and control (Lorber 2001; Yodanis 2004). These categories of difference underpin many forms of inequality, making them indispensable concerns to strategies to realise social justice.

Research on urban transformations to some extent recognises questions of racism and gender discrimination. McPhearson et al. (2016) point to the need to involve marginalised perspectives, such as those of the Black Lives Matter movement. Hamann and April (2013) explore racial segregation in Cape Town. However, the field as a whole generally avoids a strategic engagement with discrimination and inequality. Very few proposals engage with the structural drivers of inequality, the constitution of exclusionary spaces in cities and settlements, or the racialised and gendered nature of such exclusions. Topics that largely are missing from the field include, for example, the situation of refugees, displaced populations, or different forms of violence. Overall, urban sustainability transformations research does not challenge the social categories that maintain difference; instead, some proposals may reinforce inequalities (for example through infrastructure investments that reproduce environmental injustice). It is not that other diagnoses do not exist, but that they rapidly become marginalised by well‐known arguments that gain salience by their very virtue of being well known. In particular, this occurs as urban transformations become linked to research that brings concrete skill‐sets, which appear to generate practical recommendations and ways forward.

### Alignment with Pre‐Existing Notions of Progress

The urban transformations literature acts as an ivy discourse by articulating research objectives along established ideas of progress. Here, transformations theory resurrects teleological missions of societal improvement, which replicate past and present power relations into the future. That is, the formulation of research objectives within dominant paradigms of thought leads to a continued attachment to current notions of social advance.

A putative objective of transformations research is to challenge the value systems that maintain ecological degradation and social inequality. The literature emphasises the need to examine self‐reinforcing elements of systems (Nevens et al. 2013) and path dependencies that prevent change (Iwaniec et al. 2019). There is recognition of the constraints of existing institutions, lock‐ins, and “cultural” barriers to change (Castán Broto et al. 2019). However, such concerns tend to overlook dominant discourses on progress and development, such as the structure of the world economy, amassed wealth, logics of investment, and policies for perpetual growth. Programmes of action to address these issues—redistribution of wealth, community ownership, recognition of informal economies, and ways of doing business away from profit‐maximising rationales—are not central to urban transformations agendas.

Instead, several branches of the urban sustainability transformations literature successfully advance the tenets of dominant economic agendas. For example, the establishment of eco‐innovation clusters (Block and Paredis 2013; Ernst et al. 2016) and development of ICT technology (Ibrahim et al. 2018) are portrayed as driving urban transformations. There is also an interest in smart energy systems (Erlinghagen and Markard 2012; Ibrahim et al. 2018) and technological solutions that can be branded and exported (Williams 2016). These and similar studies demonstrate a faith in innovation, entrepreneurship, and investment as central to urban transformations—activities known as fundamental to the strengthening of local economies. Through this focus, the literature replicates an argument at the core of sustainability debates (and of development studies and public policy)—namely, that economic expansion is a prerequisite for social improvement. The alignment between urban growth policy and urban transformations research can be problematic for many reasons. For example, while adoption of innovative technologies is made to represent steps towards socio‐technical reconfigurations, such measures tend to benefit the current industrial system, support consumption, and enable ways of life that depend on continued resource extraction (e.g. Higgins 2013). In addition, urban development models fixed in “world city ideals” and neoliberal policies are known to drive the deteriorating conditions for the urban poor, the enclosure of commons and privatisation of assets, the homogenisation and commodification of life, gentrification, and displacement (Daher 2013; McDonald 2012; Miraftab et al. 2015).

The alignment of an ivy discourse with economic interests invites a range of actors to adopt the concept. For example, green transformations resonate with projects of state‐led economic revival (European Commission 2021; UN News 2021), beckoning as a means to re‐legitimise new forms of green statism (Luke 2009). The appeal to business is obvious: urban transformations open up a potentially profitable space for innovation and investment (e.g. World Economic Forum 2021). Other actors follow in the wake of public authorities and tech corporations (entrepreneurs, consultants, international organisations, professional associations), enticed by the promise of socially beneficial innovation. In this way, the discourse becomes firmly embedded in the global political economy. This mechanism of ivy discourses most clearly demonstrates the notion of a mobile frontier of demands, as suggested by Laclau’s (2005) floating signifiers. New sets of interests are articulated around a radical demand for change, resulting in the weight of opposing hegemonic projects being imposed upon that claim. Transformations discourse never fully articulates a chain of equivalent demands to address the dislocating effects of the environmental crisis, or to provide an alternative for action that can truly challenge the dominance of green statism perspectives.

The urban transformations literature also reproduces hegemonic norms by orienting processes of change towards pre‐established endpoints. An example is the widespread adoption of sustainability as the goal of urban transformations (Block and Paredis 2013; Ernst et al. 2016; Frantzeskaki et al. 2017; Gorissen et al. 2018; Hamann and April 2013; Ibrahim et al. 2018; Krellenberg et al. 2016; McCormick et al. 2013; McPhearson et al. 2016; Nevens et al. 2013; Pickett et al. 2013; Trencher et al. 2013; Wamsler 2015). Sustainability is a contested concept with a nearly inexhaustible range of definitions. The concept can be wielded for empowerment and recognition; however, mainstream interpretations are associated with techno‐economic rationalities, incrementalism, and cementation of the status‐quo (Castán Broto and Westman 2019). Considering the dominant themes of urban transformations research outlined above, sustainability objectives appear to materialise in transformations debates according to an established tendency to highlight environmental concerns (mainly eco‐efficiency), while marginalising social wellbeing and equality.

In contrast, decolonial scholars call for knowledge perspectives that, rather than examining the life conditions of the subaltern, develop knowledge from a subaltern perspective (Grosfoguel 2011). Knowledge is central to an “entangled package” through which structures of power are embedded in class and labour relations, racialised spaces, heteronormative conventions, and multiple other forms of inequalities expressed in everything from relationships of serfdom to aesthetic perceptions (Grosfoguel 2011). Environmental destruction and social inequalities are themselves entangled with capitalist exchanges, which produce disparities of wealth across and within nations through accumulation by dispossession (Amin 2010), and, within cities, patterns of poverty, precarity, and exclusion (Hodkinson 2012). At the same time, decolonial thinking calls against macro‐histories seen through the prism of European theoretical thought that do not challenge the symbolic sustenance of the structural apparatus of capitalism (Ndlovu‐Gatsheni 2018). Theories of urban transformations reproduce macro narratives of socio‐ecological change that hardly challenge the structural drivers of capitalism and its manifestation in specific locales, particularly, the multi‐scalar adjustments to urban life that such transformations will entail (Ajl 2015). Instead, there is a disconnect between the literature on urban sustainability transformations and the social struggles that result from these exclusions. There is recognition of the need to focus on disadvantaged populations (Henrique and Tschakert 2021; Rosenzweig and Solecki 2018). Yet, poverty and low incomes, homelessness, work precarity, serfdom, or vulnerability of migrant workers, are rarely at the centre of urban transformations debates, let alone recognition of people enduring those unequal relations as something other than passive, vulnerable groups. The rallying cries for resistance against the inequitable impacts of a globalised economy driven by social movements are also mostly absent in urban transformations discourse.

### Cementation of Epistemic Sources of Authority

Urban sustainability transformations research operates as an ivy discourse through its tendency to consolidate epistemic sources of authority. This relates to path dependencies inherent in academic knowledge production. As knowledge production is (and is meant to be) accumulative, researchers build on what they have read (and published) and what they understand to be authoritative. As a result, they insert existing theories into nascent scholarly debates (as we have done in this article). Through this process, a new concept rapidly becomes linked with frameworks in numerous disciplines. Well‐established theories are most influential, leading to replication of epistemological power imbalances.

The urban transformations literature has a tendency to universalise experiences of cities in the global North. Cities in Europe and North America have traditionally been over‐represented in urban transitions research (Romero‐Lankao and Gnatz 2013). There is a strong interest in decision‐making and planning processes within municipal government (Block and Paredis 2013; Ibrahim et al. 2018; Rijke et al. 2013; Uyarra and Gee 2013; Wamsler 2015; Williams 2016), which predominantly represent socio‐political settings of OECD nations. However, the problem is not only the location of case studies, but also that popular theories reflect the socio‐political settings in which they were derived. For example, there is a tendency to draw on governance theories developed in North America and Europe. Proposals for political and governance reform includes networked governance (Block and Paredis 2013), deliberative policymaking (Koch et al. 2016), multi‐stakeholder collaboration (Frantzeskaki and Rok 2018; Hansjürgens et al. 2018; McCormick et al. 2013; Mendizabal et al. 2018; Olazabal and Pascual 2016), multi‐level governance (Hodson et al. 2017), partnerships and intermediaries (Hamann and April 2013; Vergragt and Brown 2010), and policy entrepreneurship (Block and Paredis 2013). While these concepts can be (and indeed are) applied in different settings, they carry ideological baggage, such as the focus on formal institutions, liberal economies, contractual relations, or discrete state‐private‐civil society sectors. Such frameworks may advance assumptions linked to Eurocentric thought traditions, which make them inappropriate in other contexts (e.g. Huang et al. 2021).

The urban transformations literature has a pronounced interest in diverse forms of knowledge production, as “sustainable urban transformation involves … integrating different perspectives and bodies of knowledge and expertise” (McCormick et al. 2013:4). Research highlights knowledge co‐creation (Elmqvist et al. 2019; Trencher et al. 2013), co‐production (Frantzeskaki and Rok 2018; Iwaniec et al. 2019), co‐learning (Wiek and Kay 2015), and generation of collective imaginations (Ibrahim et al. 2018; Nevens et al. 2013). Much urban transformations research has focused on collaboration between universities and public/private organisations or between innovative forerunners in transition arenas. This can possibly strengthen dominant centres for knowledge production, but also involves nuanced accounts of power relations inherent in collective knowledge production (Frantzeskaki and Rok 2018). However, when the literature seriously engages subaltern perspectives, for example through the influence of the philosopher Achille Mbembe, scholars have found themselves questioning the putatively progressive interpretations of transformations (Schipper et al. 2021).

According to decolonial writers, a broader challenge of social justice is to understand how knowledge systems become validated, as different forms of knowing are ignored, appropriated, or instrumentalised. Quijano (2007:169) argues that one of the most durable expressions of post‐colonial power is the appropriation of imaginations. The influence of Eurocentric ideals over values can be understood as a colonial matrix of power—a superstructure that evolved over 500 years into a logic that orders all aspects of our lives today (Mignolo 2017). Non‐Western forms of knowledge have, in parallel, been systematically devalued and designated as particularistic (rather than universal) and non‐scientific (Said 1978). Santos (2015) proposes to tackle global epistemicide by embracing a plurality of ways of knowing and experiencing the world. Such effort entails active deconstruction of hegemonic vocabularies and practices that “negate, disavow, distort and deny knowledges, subjectivities, world senses, and life visions” (Mignolo and Walsh 2018:4).

While there are emerging calls for transformations research to increase the dialogue with multiple knowledges (Apgar et al. 2015; Nightingale et al. 2020), a commitment to deconstructing knowledge hegemonies is largely missing. So far, urban sustainability transformations studies overlooks a diversity of forms of knowing. A review of the literature conducted by Wolfram et al. (2016) identified seven epistemologies informing the field, out of which six were fixed in traditional scholarly disciplines (urban change theory, urban‐systems interactions, systems change, urban metabolism, resilient communities, innovation for green economies). The majority of research at that time was based on systems perspectives, revealing their strong influence. While the epistemology of grassroots innovation seemingly holds the potential to integrate activist thinking, the emphasis so far has been on urban living labs and experimentation (Wolfram et al. 2016). Studies on these two topics draw attention to new practices, up‐scaling and embedding innovations, or building legitimacy of policy processes (Frantzeskaki et al. 2016; Gorissen et al. 2018), with less attention to diverse forms of political activism, local and indigenous knowledges, and forms of community mobilisation that shape cities through a variety of purposeful and non‐purposeful processes.

The challenge of knowledge pluralism also relates to the ability to engage with individual experiences. Feminist writers call for embodied knowledge that situates action within a set of human and non‐human relations that structure how we understand justice or power (Bawaka Country et al. 2016). Urban sustainability transformations research often fails to build on situated experiences and instead displays an interest in supposedly transferable frameworks and roadmaps (Ibrahim et al. 2018; Li et al. 2018; Pickett et al. 2013). In fact, a surprising feature of the literature is the invisibility of individuals. Overall, personal narratives are absent from the field, as well as emotions (suffering, frustration, joy, or loss) that may inspire resistance or change. Personal change is conceived as a generic, cultural change involving everyone (O’Brien 2018). To the extent that individuals feature, it is often in their role as consumers. Here, they are important insofar as they absorb new behaviours conducive to lower ecological footprints. For example, “creating visions of sustainable lifestyles” becomes an “imperative to the design and governance of more sustainable cities” (McCormick et al. 2013:4). Barriers to behavioural change are similarly presented as factors that prevent transformations (Mendizabal et al. 2018). These perspectives draw attention away from experiential accounts that could describe problems and inspire directions for change.

## Conclusions

In this paper, we engage with urban transformations theory as a case study of ivy discourses. The focus on cities provides concrete illustrations of how transformations theory is applied, in relation to specific issues and through its encounter with urban politics. However, urban transformations is only one manifestation of the operation of transformations discourse. The traction of transformations as an academic buzzword (Davis 1986; Davis 2008) and as a concept that is open to appropriation (Castán Broto and Westman 2019), suggests that the discourse has the capacity to operate as an ivy discourse.

Our concern is whether transformations discourse, by acting as an empty signifier, can generate radical potential? This could be achieved by its ability to navigate epistemic boundaries, enabling the development of alternatives that support the wellbeing of people across the world. Is transformation a parallel concept to sustainability, with the potential to engage with the future as a means to bring current environmental struggles firmly into the realm of politics (Brown 2016)? The concept of transformations operates differently, because it accommodates different demands into hegemonic discourses rather than challenging them and producing alternatives. Back to Laclau’s (2005) ideas of hegemonic imposition, urban transformations discourse effectively articulates new links around radical demands for societal change, gradually obscuring a plurality of struggles in favour of cemented ideas and interests.

The problem with ivy concepts is that the roots of radical thought remain (barely) visible beneath an impressive conceptual apparatus, which ensures the continual attraction of researchers and policymakers. This distracting overgrowth is usually not directly dangerous or destructive—it simply rehashes what is already known, which prevents the thriving of new ideas. Meanwhile, the discourse parasitically builds on emancipatory thought without advancing it. In doing so, it displaces theoretical alternatives that, rather than aiming at creating a grand theory or dogma to direct future action, engage with situated, affective experiences that surprise and produce wonder. These alternatives are only validated in their practical implementation, as advocated in the idea of minor theory (Katz 1996) or Santos’ (2015) idea of rearguard theory. In contrast, transformations is attractive because it matches a definition with a direction, something central for the kind of dogmatic thinking that Santos (2015) warns against.

The drive to examine how academic theories relate to dominant discourses is inspired by the statement that “[w]e do not need alternatives so much as we need an alternative thinking of alternatives” (Santos 2015:42). How can we generate alternatives from within the theoretical and ideological traditions that resulted in the problems that we face in the first place? We suggest through this paper that scholars that engage with radical concepts may unwittingly contribute to the reproduction of hegemony. Following Laclau and Mouffe (2001), discourses of action are transversed by the contingent conditions in which they emerge. This does not imply that all of academia is rooted in discursive‐ideological reproduction or is complicit with groups in power. As we have shown, scholarly debates occupy a range of ideas, including proposals that directly threaten dominant interests, advance incremental change, and conform with existing social relations. But, as explained by the concept of ivy discourses, theories that find resonance in dominant disciplines, that align with political‐economic constellations, and reinforce epistemic sources of authority are those that most readily ripple through academia and the rest of society.

Such insights raise uncomfortable questions regarding our ability, as academic‐activists, to support radical thinking and doing. Rather than focusing on ivy discourses, current scholarship could turn to a greater extent to on‐the‐ground action for justice, building on the rich global heritage of environmental activism (Agyeman et al. 2016; Martinez‐Alier et al. 2016; Temper et al. 2018). Yet, it is also true that some academic theories have exceptional force in society, whether intended or not. As we have argued elsewhere (Castán Broto and Westman 2019), concepts that have been appropriated remain open to re‐appropriation for progressive means. Ultimately, what defines the radical potential of any social theory is the ways in which it is deployed. Urban transformations discourse can act as a vehicle for emancipatory thought, but only if it remains autonomous from the kind of vanguard theorising that has already explained the world before even engaging with it. Whether or not this will be possible depends on the degree to which we can be reflexive about inequalities and power relations embedded in academia itself.

## Data Availability

Data sharing is not applicable to this article as no new data were created or analyzed in this study.
